# Modification of Pea Starch and Dextrin Polymers with Isocyanate Functional Groups

**DOI:** 10.3390/polym10090939

**Published:** 2018-08-23

**Authors:** Reza Hosseinpourpia, Arantzazu Santamaria Echart, Stergios Adamopoulos, Nagore Gabilondo, Arantxa Eceiza

**Affiliations:** 1 Department of Forestry and Wood Technology, Linnaeus University, Lückligs Plats 1, 35195 Växjö, Sweden; stergios.adamopoulos@lnu.se; 2 Materials + Technologies’ Group, Department of Chemical & Environmental Engineering, Polytechnic College of San Sebastian, University of the Basque Country UPV/EHU, Pza. Europa 1, 20018 Donostia-San Sebastián, Spain; arantzazu.santamaria@ehu.eus (A.S.E.); nagore.gabilondo@ehu.eus (N.G.)

**Keywords:** diisocyanate, DVS, pea starch, dextrin, functionalization, unequal reactivity

## Abstract

Pea starch and dextrin polymers were modified through the unequal reactivity of isocyanate groups in isophorone diisocyanate (IPDI) monomer. The presence of both urethane and isocyanate functionalities in starch and dextrin after modification were confirmed by Fourier transform infrared spectroscopy (FTIR) and ^13^C nuclear magnetic resonance (^13^C NMR). The degree of substitution (DS) was calculated using elemental analysis data and showed higher DS values in modified dextrin than modified starch. The onsets of thermal degradation and temperatures at maximum mass losses were improved after modification of both starch and dextrin polymers compared to unmodified ones. Glass transition temperatures (*T*_g_) of modified starch and dextrin were lower than unmodified control ones, and this was more pronounced in modified dextrin at a high molar ratio. Dynamic water vapor sorption of starch and dextrin polymers indicated a slight reduction in moisture sorption of modified starch, but considerably lower moisture sorption in modified dextrin as compared to that of unmodified ones.

## 1. Introduction

Starch, as the main reserve source and energy storage of plants, is widely available in agricultural products. This biopolymer has excellent applications in many industrial sectors such as food, textile, pharmaceutical, paper, and biofuel. Most of these applications rely on the colloidal properties of two structurally distinct α-d-glucan components of starch granules, amylose, and amylopectin [1]. Amylose is an essentially linear macromolecule with mostly α(1–4)-linked d-glucopyranosyl units and less than 0.5% of the glucose residues in α(1–6) linkages. Amylopectin is larger than amylose with α(1–4) linked chains and up to about 5% of the glucose residues in α(1–6) branch units [2]. Starch from different sources has different amylose/amylopectin ratios, and this, in turn, has a great effect on thermal behavior, mechanical strength and also accessibility of the starch granules to water molecule and other chemical reagents [3,4].

Over the last decades, starch has been modified chemically, physically, genetically, and enzymatically to enhance its functionalities and offer new applications. Chemical modification of starch has been carried out through cross-linking, acid hydrolysis, oxidation, etherification, esterification, cationization, and grafting of polymers [4,5,6,7,8,9,10,11,12]. These reactions partially or entirely destruct the hydrogen bonds of the starch and induce distinct changes in the properties of the ensuing polymers. Some examples of chemically modified starch are biodegradable packaging materials, thermoplastic starch (TPS), and thin films with improved mechanical and thermal properties. 

However, modification of starch polymers with isocyanate functional groups has received very little attention. Barikani and Mohammadi [5] grafted polycaprolactone terminated hexamethylene diisocyanate (HDI) onto the hydroxyl groups of corn starch polymer. Modified starch showed higher hydrophobicity than unmodified. Modification of starch nanoparticles with HDI also increased the hydrophobicity of thin films and enhanced their thermal stability and electrical conductivity [9]. Cross-linking of corn starch with a polypropyleneoxide toluene diisocyanate (PTD) oligomer at ambient temperature led to the production of amorphous elastomeric networks with reduced swelling capacity in different solvents due to the formation of polyurethane linkages [13]. The DSC curves showed an absence of glass transition temperature (*T*_g_) in unmodified starch but two defined *T*_g_ signals at −60 and 35 °C were obtained, which were respectively attributed to the relaxation of the grafted oligopropylene oxide chains and possible glass transition of the starch macromolecules in the network. 

Isophorone diisocyante (IPDI) is an aliphatic isocyanate that contains primary and secondary isocyanate groups with unequal reactivity. Modification of cellulose nanocrystals with IPDI monomer facilitated the covalent bonding between the modified cellulose and polymer matrix, and brought a greater mechanical strength than that of unmodified cellulose nanocrystals [14]. Gómez-Fernández et al. [15] functionalized a kraft lignin with IPDI for producing polyurethane flexible foams. It was reported that the modification of lignin contributed to a higher chemical bonding of the lignin to the polyurethane matrix and also to the production of more soft and flexible foams than unmodified lignin containing foams. Reactivity of the secondary and primary isocyanate groups of the IPDI isomers increases by using a proper catalyst. Lewis acids such as dibutyltin dilaurate (DBTDL) preferentially enhance the reactivity of the cycloaliphatic (secondary) isocyanate group, while Lewis base like trimethylamine or 1,4-diazabicyclo[2.2.2]octane (DABCO) catalyzes the reaction of the primary isocyanate group [16,17,18,19].

Enhancement of the unequal reactivity of IPDI isocyanate groups by using DBTDL catalyst should make possible a reaction with the functional groups of natural polymers, like the hydroxyl groups of starch, through their higher reactive sites (e.g., –NCO groups connected to the rings). A pendant isocyanate group, which is sterically hindered by the neighboring methyl group, should then be available for reaction with additional monomers of interest. Therefore, this study aims at confirming the hypothesis by studying the highly functionalized pea starch and dextrin polymers with IPDI monomer and DBTDL reaction catalyst. Characterization of the modified starch and dextrin involved elemental analysis, Fourier transform infrared spectroscopy (FTIR), ^13^C nuclear magnetic resonance (^13^C NMR) in solid state, thermogravimetric analysis (TGA), differential scanning calorimetry (DSC), and scanning electron microscopy (SEM). The effect of functional modification on water vapor sorption behavior of modified samples was assessed by using a dynamic vapor sorption (DVS) apparatus.

## 2. Materials and Methods

### 2.1. Materials

Pea starch and pea dextrin were kindly provided by Emsland-Stärke GmbH (Wietzendorf, Germany). The functionalization of starch and dextrin polymers with isocyanate groups was carried out using isophorone diisocyanate, IPDI (>99.5%, DesmodurI^®^, kindly provided by Covestro, Leverkusen, Germany), with a NCO content of 37.8%. HPLC grade anhydrous dimethyl sulfoxide (DMSO) (>99.8%, Macron Fine Chemicals, Paris, KY, USA) was used as reaction medium and dibutyltin dilaurate DBTDL (>95%, Sigma Aldrich, Saint Louis, MO, USA) catalyzed the reaction of hydroxyl groups from glucoside unities of starch and dextrin with the secondary NCO groups of IPDI. HPLC grade toluene (>99.8%, Lab-Scan Analytical Sciences, Gliwice, Poland) and tetrahydrofuran (THF) (>99.8%, Macron Fine Chemicals) were used to wash the polymers and remove the unreacted IPDI after modification.

### 2.2. Functionalization of Starch and Dextrin

1 g of vacuum oven dried-starch was dissolved in 70 mL of DMSO at 90 °C by magnetic stirring for 120 min. The same concentration of dextrin was added to DMSO and dissolved at 40 °C by magnetic stirring for 120 min. A separate 3-neck flask partially submerged in an oil bath containing IPDI was heated to 60 °C under a flowing nitrogen atmosphere. The DBTDL (0.1% of the total weight) was then added to the IPDI and allowed to stir for 5 min. The dissolved starch was added dropwise to the stirring IPDI/DBTDL mixture with a separatory funnel over 45 min while maintaining the vigorous stirring in the flask. The reaction proceeded at 60 °C for 24 h in a flask with N_2_ inlet. The reaction was run in excess of IPDI at a weight ratio of NCO:OH of 3:1 and 6:1. The reaction was halted by addition of THF. The DMSO and THF were then removed by 3 times centrifugation at 4500 rpm for 5 min replacing the supernatant by fresh THF after each centrifugation. The precipitates were then further washed with toluene and centrifuged at 4500 rpm for 10 min, and repeated 3 times, followed by replacing the used toluene with the fresh one after each centrifugation in order to remove the remaining unreacted IPDI. An identical procedure was applied to produce the modified dextrin. Finally, the modified starch at different ratios, namely MS 3.1 and MS 6.1, as well as the modified dextrin, called MD 3.1 and MD 6.1, were dried in a vacuum oven at 50 °C for 24 h, and then the vacuum was kept at room temperature for the further 24 h. The samples were then stored in a tight bottle.

### 2.3. Characterization

#### 2.3.1. Elemental Analysis

The carbon, hydrogen, oxygen and nitrogen contents of unmodified and modified starch and dextrin were determined by elemental analysis using an EuroVector EA 3000 atomic absorption spectrometer (Pavia, Italy) heated at 980 °C with a constant flow of helium.

#### 2.3.2. Fourier Transform Infrared Spectroscopy

The chemical structure of the unmodified and modified starch and dextrin as well as of the IPDI monomer was analyzed with FTIR (Nicolet-Nexus, Waltham, MA, USA) provided with a MKII Golden Gate accessory (Specac, Orpington, UK) with a diamond crystal at a nominal incidence angle of 45° and ZnSe lens. Spectra were recorded in attenuated reflection (ATR) mode and the evaluation was performed between 4000 and 400 cm^−1^ at room temperature, averaging 64 scans with resolution of 4 cm^−1^. The samples were dried at 50 °C for 24 h prior to the analysis.

#### 2.3.3. Nuclear Magnetic Resonance Spectroscopy

The insertion of isocyanate functional groups in starch and dextrin polymers were verified with solid-state ^13^C NMR using a Bruker Avance III 400 MHz equipment (Billerica, MA, USA). The spectra were recorded using a decoupled sequence at ^13^C frequency of 100.6338 MHz at 25 °C. Samples were measured at a spinning rate of 10,000 Hz averaging 4096 scans with a recycling delay of 10 s. A time domain of 2 K was employed with a spectral width of 30 KHz.

#### 2.3.4. Thermogravimetric Analysis

Thermal stability of the modified starch and dextrin was analyzed using a Q500 TA equipment (New Castle, DE, USA). Around 5 mg of dried sample (24 h at 50 °C) were heated from 50 to 650 °C at a rate of 10 °C·min^−1^, under a flowing nitrogen atmosphere.

#### 2.3.5. Differential Scanning Calorimetry

The changes in glass transition temperature (*T*_g_) of starch and dextrin associated with functional modification with IPDI were performed using a DSC analyzer (Mettler Toledo DSC3+ equipment, Columbus, OH, USA), from −50 to 200 °C at a heating rate of 30 °C min^−1^ under a nitrogen flow of 10 mL·min^−1^. Approximately 5 mg of oven-dried sample (at 50 °C for 24 h) was used for each analysis.

#### 2.3.6. Dynamic Vapor Sorption

The vapor sorption behavior of the modified polymers was determined using a DVS apparatus (Q5000 SA, TA Instruments, New Castle, DE, USA) as reported previously [20,21,22]. Approximately 8 mg of oven-dried (at 50 °C for 24 h) modified and control starch and dextrin granules were used for each measurement. The relative humidity (RH) increased from 0 to 90% in step sequences of 15% and then 5% from 90 to 95% RH. The instrument maintained a constant target RH until the mass change in the sample (dm/dt) was less than 0.01% per minute over a 10 min period. The target RH, actual RH, sample mass and running time were recorded every 30 s during the sorption run. The moisture content of the granules was calculated based on their equilibrium weight at each given RH step throughout the sorption run measured by the DVS device.

#### 2.3.7. Scanning Electron Microscopy

The morphology of the starch and dextrin granules before and after modification was characterized by SEM using a JEOL JSM-7000F equipment (Akishima, Japan) operating at 20 kV and a surrounding beam current between 0.01 and 0.1 nA, taking secondary electron images. Dried samples were placed over a double-sided carbon-based conductive tape and covered with a 20 nm Au in order to make the surface conductive.

## 3. Results and Discussion

### 3.1. Elemental Analysis and Degree of Substitution (DS)

The results of elemental analysis and DS of the unmodified and modified starch and dextrin are given in Table 1. These results indicate a negligible nitrogen content of unmodified starch (S-control) and dextrin (D-control), but considerable amounts of nitrogen were found in modified samples. The content of nitrogen in MS 3.1 and MS 6.1 are 5.28% and 6.24%, respectively, and respective amount of nitrogen in MD 3.1 and MD 6.1 are 7.38% and 8.12%, respectively. The enhanced nitrogen contents of modified starch and dextrin suggested that IPDI was successfully substituted with the hydroxyl groups of polymers’ backbones.

Although the molar ratio of IPDI in reaction medium of MS 6.1 was two times more than MS 3.1, the DS value of MS 3.1 (0.55) was slightly lower than that (0.77) of MS 6.1. This might be explained by the different reactivity of the three –OH groups in a glucose unit of the starch macromolecule. The primary C_6_ OH is more reactive and readily accessible than the secondary ones on C_2_ and C_3_. The C_2_ OH is however more reactive than the C_3_, mainly because the former is closer to the hemi-acetal and more acidic than the latter [23]. The hydroxyl groups in the dextrin polymer showed higher reactivity than those in the starch polymer, and thus the DS values of MD 3.1 and MD 6.1 were 1.15 and 1.52, respectively. This might be due to the higher solubility of dextrin in solvent than starch, and also to the low availability of starch’s hydroxyl groups due to the formation of gel-like product during the modification reaction. A more detailed description regarding the calculation of DS can be found in the Supplementary Materials.

### 3.2. Structural Characterization

**Figure 1:**
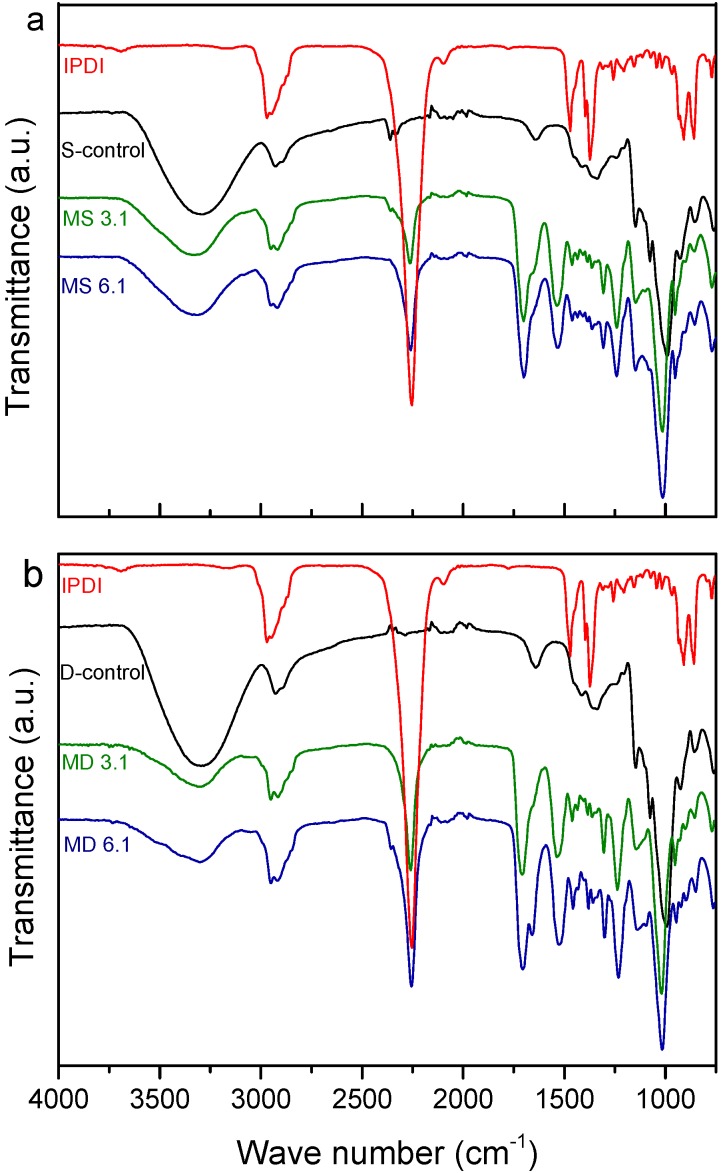
Fourier transform infrared spectroscopy (FTIR) spectra of IPDI, S-control, MS 3.1 and MS 6.1 (**a**), and IPDI, D-control, MD 3.1 and MD 6.1 (**b**).

FTIR spectroscopy analysis was used to monitor changes in the structures of starch and dextrin polymers upon functionalization with the IPDI monomer. Figure 1a,b illustrates the FTIR spectra of unmodified and modified starch and dextrin as well as of IPDI monomer. There were no differences in the FTIR spectra of unmodified starch and dextrin, as they showed peaks at 3450 cm^−1^, which is assigned to the stretching vibration of –OH group, and at 2960 cm^−1^, which can be attributed to –CH bond stretching vibration [24,25]. The absorption band between 1000 and 1200 cm^−1^ was characteristic of the –CO stretching of the polysaccharide skeleton [9]. Obvious changes across all regions of the spectra were observed for the modified starch and dextrin. There was an apparent decrease in the –OH peak of modified starch and dextrin at 3450 cm^−1^, which indicated that many of these functional groups were consumed. This together with the appearance of the isocyanate band at 2266 cm^−1^, which is related to the pendant –NCO groups, indicates the success in functionalization starch and dextrin with IPDI [14,15,19]. After modification, the modified polymers were washed with toluene three times. The FTIR spectra of the third centrifuged product (see Figure S1) were almost comparable with the spectra of the neat toluene, which indicates that the –NCO peaks in modified starch and dextrin can be attributed to the attachment of isocyanate groups into the starch and dextrin polymers. In addition, increase in the absorbance of –CO groups at 1650–1750 cm^−1^, and bending vibration of –NH and stretching vibration of C–N at 1530 cm^−1^ confirmed the presence of urethane bonds between IPDI and starch and dextrin [14,15,26].

**Figure 2:**
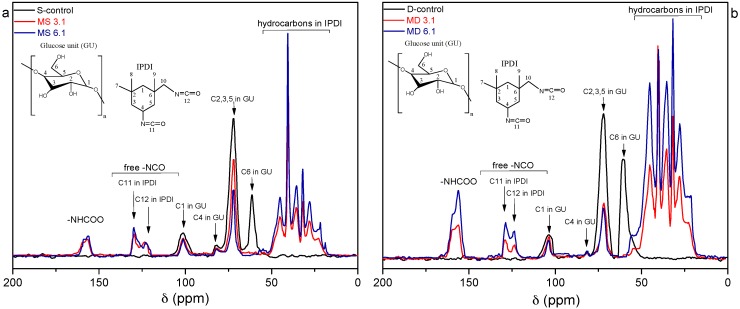
^13^C nuclear magnetic resonance (^13^C NMR) spectra of S-control, MS 3.1 and MS 6.1 (**a**) and D-control, MD 3.1 and MD 6.1 (**b**).

The structural changes of starch and dextrin polymers after modification were analyzed by means of solid NMR. Figure 2a,b illustrate almost identical spectra for unmodified starch and dextrin, although unmodified dextrin showed a more intense signal at 61.8 ppm, which is assigned to C_6_ [27,28]. The overlapping signal at around 68–78 ppm is collectively associated with C_2_, C_3_, and C_5_ [2,29]. Glycosidic carbons C_1_ and C_4_ profiles are most sensitive to chain conformations [30], and therefore the resonances that appear at 81.4 and 103.2 ppm are due to the amorphous domains of the C_4_ and C_1_, respectively [2,31]. Modification of starch and dextrin with IPDI caused apparent changes on NMR spectra, and the intensity of signals was more pronounced at the high NCO:OH ratio (6:1). Appearance of new peaks between 20 and 50 ppm corresponded to hydrocarbons that are present in the structure of isocyanate; in detail, the signal at 45.2 ppm is associated with the methylene group in the aliphatic IPDI ring, peaks at 26.9 and 32.3 ppm are attributed to the tertiary carbons, and signals at 27.8 and 23.5 ppm are due to the methyl groups [14]. The appearance of new peaks between 120 and 160 ppm correspond to urethane (155.2–158.0 ppm) and –NCO (122.2–129.5) linkages in the starch and dextrin polymers [14,15]. The observation of distinct peaks for the primary urethane linkage can be attributed to the existence of excess IPDI, which may cause some side reactions with the OH groups of starch and dextrin polymers. The primary free –NCO group appears at 123.6 ppm and the secondary free –NCO group is present at 129.5 ppm. The availability of free primary and secondary –NCO groups in the starch and dextrin polymers induce them as remarkable bio-macromolecules for further reaction with other polymer matrices. The NMR results together with the FTIR and elemental analysis data, confirmed that the starch and dextrin polymers were highly modified with acquisition of isocyanate and urethane functionalities, and thus the hypothesis was approved.

### 3.3. Thermal Behavior

**Figure 3:**
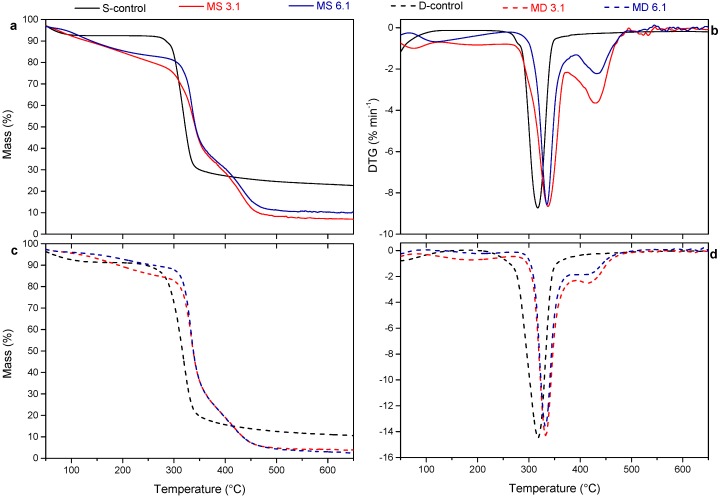
Mass loss and first derivative (DTG) of S-control, MS 3.1 and MS 6.1 (**a**,**b**) and D-control, MD 3.1 and MD 6.1 (**c**,**d**).

Thermal degradation behavior of unmodified starch and dextrin as well as of the modified ones were examined by thermogravimetric (TG) and derivative thermogravimetric (DTG) analyses of samples that were dried previously in a vacuum oven at 50 °C for 24 h. Figure 3a,c shows the temperature range that unmodified and modified starch and dextrin lost their masses, and Figure 3b,d demonstrates the peaks of DTG curves that are associated with the decomposition temperatures of samples [32]. There were considerable differences in the degradation patterns of starch and dextrin after functionalization with IPDI. Modified starch and dextrin showed higher onset temperature of degradation (*T*_onset_) than the unmodified ones. The *T*_onset_ of S-control was 297.2 °C and it was increased to 313.2 and 324.4 °C in MS 3.1 and MS 6.1, respectively. D-control showed slightly lower *T*_onset_ (292.1 °C) as compared with S-control. Modification of dextrin also increased the respective *T*_onset_ of MD 3.1 and MD 6.1 to 318.9 and 319.8 °C (Table 2). The DTG curves were derived by the slope of TG graphs (Figure 3b,d), and illustrate the first temperatures at which the maximum mass loss (*T*_max1_) of the polymers occurred. These temperatures were 316.6 and 319.7 °C for starch and dextrin, respectively, and were directly associated with their thermal decomposition [33]. As indicated in Table 2, functionalization with IPDI, in contrast, shifted the *T*_max1_ to slightly higher temperatures at ~330–338 °C, which can be attributed to the decomposition of urethane groups into the isocyanate and alcohol groups [14,34], and caused second maximum degradation temperatures (*T*_max2_) at ~413–434 °C for modified starch and dextrin. These results are in accordance with Girouard and coworkers, who found more than one *T*_max_ in cellulose nanocrystals after functionalization with IPDI [14]. Valodkar & Thakore [9] also reported an improved thermal stability of starch nanoparticles after modification with 1,4-hexamethylene diisocyanate (HMDI).

**Figure 4:**
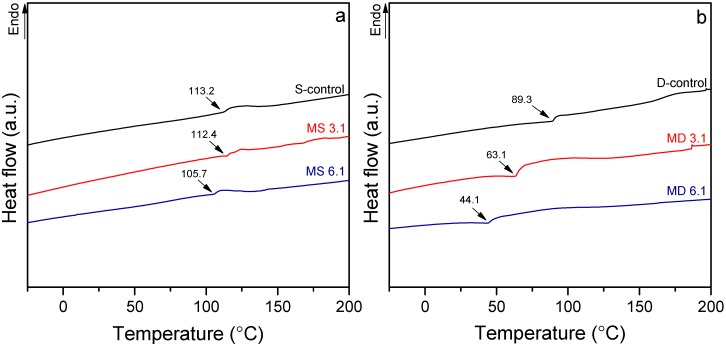
Differential scanning calorimetry (DSC) thermographs of S-control, MS 3.1 and MS 6.1 (**a**) and D-control, MD 3.1 and MD 6.1 (**b**).

The glass transition temperature (*T*_g_) of unmodified and modified starch and dextrin after removing the first history of samples are shown in Figure 4a,b. The *T*_g_ of unmodified starch and dextrin were found to be 113.2 and 89.3 °C, respectively. Previous studies quoted that the moisture content has a considerable effect on shifting the *T*_g_ of starch [35,36,37]. Thus, the lower moisture content of unmodified starch and dextrin in this study resulted to a higher *T*_g_ than former studies. The respective *T*_g_ of MS 3.1 and MS 6.1, however, shifted to 112.4 and 105.7 °C. Modification of dextrin caused further decrease in *T*_g_, as MD 3.1 presented a *T*_g_ of 63.1 °C and MD 6.1 showed a *T*_g_ of 44.1 °C. The obvious decrease in *T*_g_ of modified polymers, particularly modified dextrin, might be attributed to the insertion of IPDI in the polymers’ backbones and creation of urethane linkages, which can increase the mobility of polymers due to lower amounts of hydroxyl groups, and thus reduce the hydrogen bonds [15]. In addition, presence of the pendant isocyanate groups on starch and dextrin may hinder the packaging of polymer due to steric hindrance, and thus decrease the *T*_g_. 

### 3.4. Water Vapor Sorption Behavior

**Figure 5:**
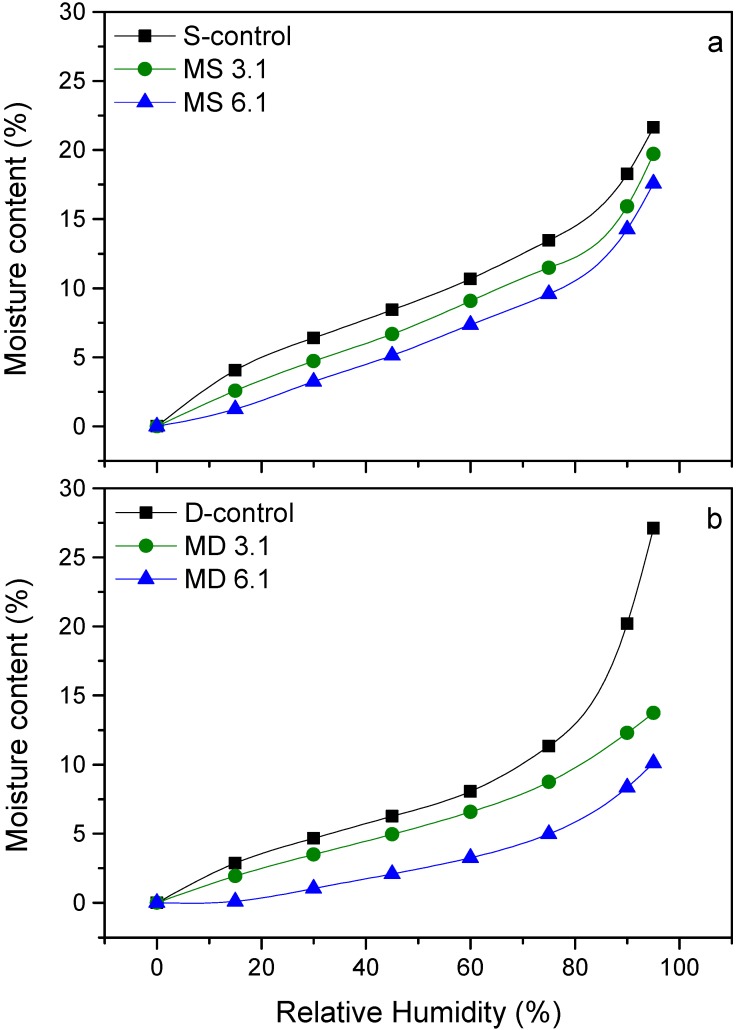
Moisture content (MC) of S-control, MS 3.1 and MS 6.1 (**a**) and D-control, MD 3.1 and MD 6.1 (**b**) for whole range of RH.

Dynamic water vapor sorption analysis showed obvious changes in moisture adsorption behavior of starch and dextrin as a result of IPDI modification (Figure 5a,b). Moisture content of samples increased with increasing the relative humidity (RH) from 0% to 95%. MS 3.1 and MS 6.1 demonstrated a comparable sorption behavior as S-control across all RH range, but with lower moisture content (MC). The upward bending of sorption curves at 75% RH might be due to the relaxation of amorphous regions of the polymers, which resulted in accommodation of more water molecules [20]. S-control exhibited a MC of 21.6%, while MC of MS 3.1 and MS 6.1 were 19.7% and 17.6%, respectively, at 95% RH. The increase in moisture content of D-control was more pronounced in RHs of above 75%, and it reached MC of 27% at 95% RH. This might be related to the high amorphous structure of dextrin polymer which eases the water vapor accommodation. Modification of dextrin with IPDI, however, strongly decreased the moisture adsorption, as the respective MCs of MD 3.1 and MD 6.1 were 13.7% and 10.1% at 95% RH. Reduction in MC of modified starch and dextrin can be due to occupation of polar hydroxyl groups by cycloaliphatic urethane moieties in the polymer chains. Similar results were reported by Barikani & Mohammadi [5] as well as by Valodkar & Thakore [9], who quoted that the hydrophilicity of starch polymer was decreased by urethane modification. These results along with the thermal analysis provided further evidence of the starch- and dextrin-urethane functionality and demonstrated the improved thermal stability and decreased moisture adsorption behavior as a result of the IPDI modification.

### 3.5. Scanning Electron Microscopy (SEM)

**Figure 6:**
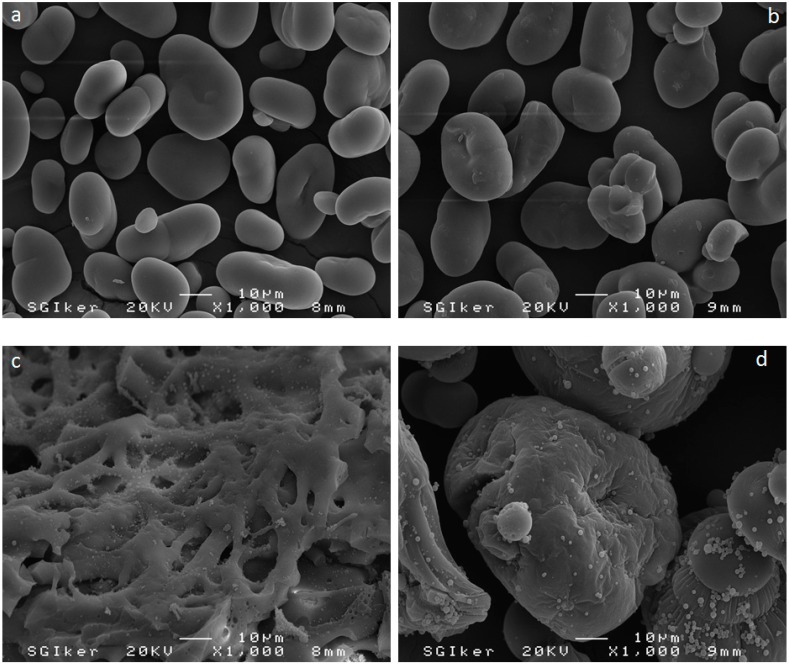
Scanning electron microscopy (SEM) micrographs of S-control (**a**), D-control (**b**), MS 6.1 (**c**), MD 6.1 (**d**).

SEM micrographs of controls and modified samples at the higher weight ratios (MS 6.1 and MD 6.1) are presented in Figure 6a–d. These images clearly show an intense allocation of spherical particles on the modified starch and dextrin granules (Figure 6c,d). It is also obvious that the smooth surfaces of unmodified starch and dextrin granules turned to entirely rough surfaces after modification with IPDI. This was reported previously by Barikani & Mohammadi [5]. In addition, size of the dextrin granules was increased after modification, while modified starch presented a more dense structure and intra-connection between granules. This might be due to the existence of excess IPDI, which resulted in possible side reactions between modified starch and dextrin granules, i.e., reaction between primary isocyanate groups with OH groups of starch and dextrin polymers. The SEM micrographs provide the visual approval on successful modification of starch and dextrin granules by IPDI. Further images from the modified samples MS 3.1 and MD 3.1 (lower mole ratios) are given in Figure S2.

## 4. Conclusions

Pea starch and dextrin were successfully modified with isophorone diisocyanate (IPDI) in order to reduce their hydrophilicity and improve their thermal stability. Assessment of degree of substitution (DS) from elemental analysis data indicated that most of the hydroxyl groups in starch and dextrin polymers reacted with IPDI. FTIR and ^13^C NMR confirmed both isocyanate and urethane functionalities in starch and dextrin macromolecules after modification. This study demonstrated remarkable improvement in thermal stability of IPDI-modified starch and dextrin. In addition, the moisture sorption of starch and dextrin was greatly reduced by IPDI modification. Functionalization of starch and dextrin polymers with IPDI make them suitable for a wide range of applications. These sustainable materials can be employed as matrices for composites or as reinforcement elements in other matrices without any further functionalization. For example, such polysaccharides would constitute promising substitutes for petroleum-based counterparts, e.g., in polyurethane foams and wood adhesive applications.

## Figures and Tables

**Table 1:** Elemental composition of unmodified and modified starch and dextrin.

	C	H	O	N	DS
S-control	38.60	6.53	52.94	0.08	-
MS 3.1	45.58	7.45	26.96	5.28	1.11
MS 6.1	46.97	7.58	27.92	6.24	1.54
D-control	39.33	6.42	52.45	0.03	-
MD 3.1	48.40	7.98	23.68	7.38	2.29
MD 6.1	53.13	8.02	24.08	8.12	3.04

**Table 2:** Thermal degradation properties of unmodified and modified starch and dextrin (°C).

	*T*_onset_	*T*_max1_	*T*_max2_
S-control	297.2	316.6	-
MS 3.1	313.2	334.7	430.4
MS 6.1	324.4	337.6	433.6
D-control	292.1	319.7	-
MD 3.1	318.9	330.1	415.1
MD 6.1	319.8	334.8	417.5

## Supplementary Materials

The following are available online at http://www.mdpi.com/2073-4360/10/9/939/s1, Figure S1: FTIR spectra of neat toluene and toluene from the third washing, Figure S2: SEM micrographs of MS 3.1 (a) and MD 3.1 (b).
