# Supplementary material for: Modification of Pea Starch and Dextrin Polymers with Isocyanate Functional Groups
Source: Polymers (Basel). 2018 Aug 23;10(9):939. doi: 10.3390/polym10090939 (PMC6403980; doi:10.3390/polym10090939)
Supplement: Supplementary file 1 [file polymers-10-00939-s001.pdf]

Supporting Information

Modification of pea starch and dextrin polymers with isocyanate functional groups

Reza Hosseinpourpia [1,*], Arantzazu Santamaria Echart [2], Stergios Adamopoulos [1], Nagore Gabilondo [2] and Arantxa Eceiza [2,*]

[1] Department of Forestry and Wood Technology, Linnaeus University, Lückligs Plats 1, 35195 Växjö, Sweden; reza.hosseinpourpia@lnu.se

[2] Materials + Technologies' Group, Chemical & Environmental Engineering Dep., Polytechnic College of San Sebastian, University of the Basque Country UPV/EHU, Pza. Europa 1, 20018 Donostia-San Sebastián, Spain; arantzazu.santamaria@ehu.eus

[3] Department of Forestry and Wood Technology, Linnaeus University, Lückligs Plats 1, 35195 Växjö, Sweden; stergios.adamopoulos@lnu.se

[4] Materials + Technologies' Group, Chemical & Environmental Engineering Dep., Polytechnic College of San Sebastian, University of the Basque Country UPV/EHU, Pza. Europa 1, 20018 Donostia-San Sebastián, Spain; nagore.gabilondo@ehu.eus

[5] Materials + Technologies' Group, Chemical & Environmental Engineering Dep., Polytechnic College of San Sebastian, University of the Basque Country UPV/EHU, Pza. Europa 1, 20018 Donostia-San Sebastián, Spain; arantxa.eceiza@ehu.eus

*Corresponding authors:

Phone: +34-943-017185 E-mail: arantxa.eceiza@ehu.es

Phone: +46-470-708074 E-mail: reza.hosseinpourpia@lnu.se

Degree of substitution (DS)

The DS was calculated according to the equation S.1, which adjusted from Wang *et al.*[1] and Heinze *et al.*[2] due to the two functional groups in IPDI:

$$DS = \frac{\frac{162.15 \times N\%}{14 \times 100 - 119.12 \times N\%}}{2} \qquad \text{S.1}$$

where N% is nitrogen content (%) determined by elemental analysis method, 162.15 is the molecular weight of anhydrous glucose unit, 119.12 is the molecular weight of IPDI, and 14 represents the nitrogen atomic mass.

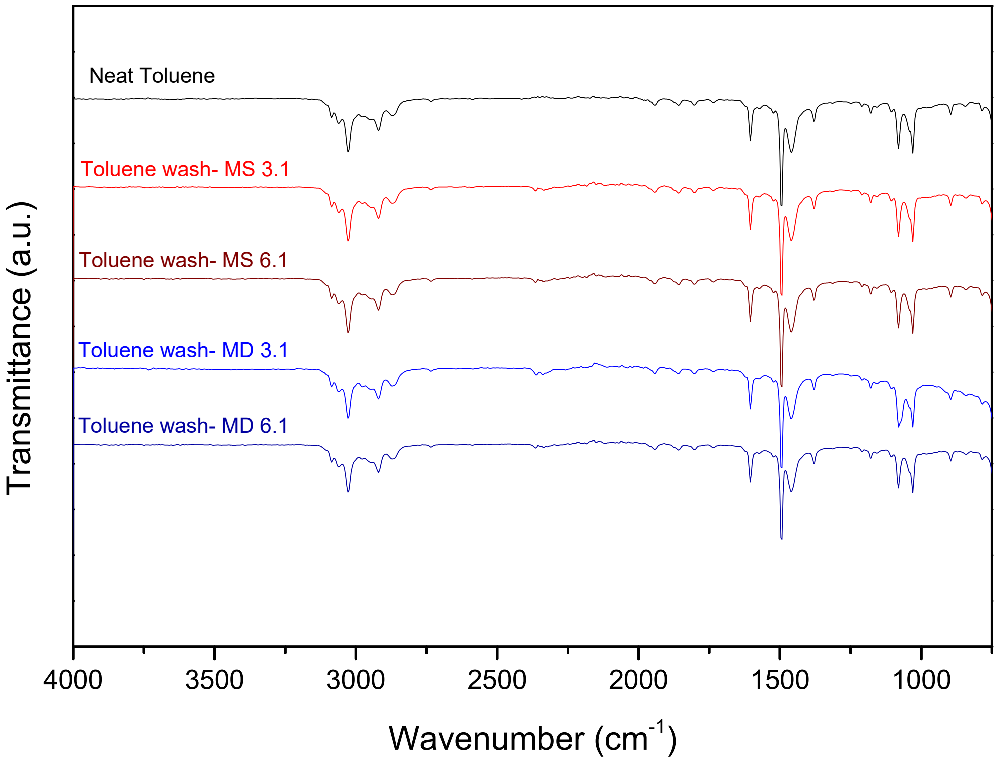

Figure S1: FTIR spectra of neat toluene and toluene from the third washing.

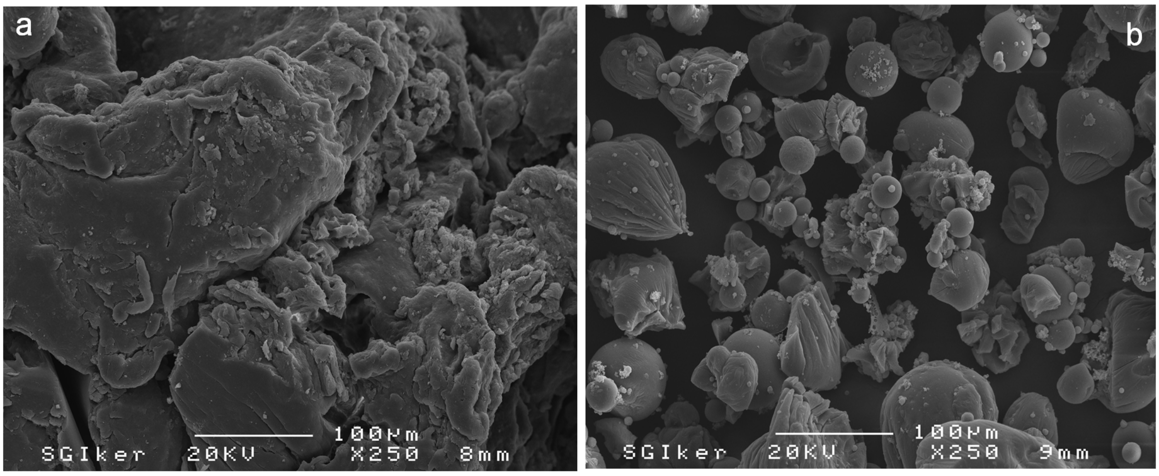

Figure S2: SEM micrographs of MS 3.1 (a) and MD 3.1 (b)

References

(1) Wang, P.; Wu, X.; Dong-hua, X.; Xu Kun, X.; Ying, T.; Xi-bing, D.; Wen-bo, L. Preparation and characterization of cationic corn starch with a high degree of substitution in dioxane–THF–water media, Carbohyd. Res.*,* **2009**, *344*, 851-855.

(2) Heinze, T.; Haack, V.; Rensing, S. Starch derivatives of high degree of functionalization. Preparation of cationic 2-hydroxypropyltrimethylammonium chloride starches, Starch-Stärke., **2004**, *56*, 288-296.
